# Modeling the global impact of reducing out-of-pocket costs for children’s surgical care

**DOI:** 10.1371/journal.pgph.0002872

**Published:** 2024-01-26

**Authors:** Emily R. Smith, Pamela Espinoza, Madeline Metcalf, Osondu Ogbuoji, Cesia Cotache-Condor, Henry E. Rice, Mark G. Shrime

**Affiliations:** 1 Department of Emergency Medicine, Duke University School of Medicine, Durham, North Carolina, United States of America; 2 Duke Center for Global Surgery and Health Equity, Duke University School of Medicine, Durham, North Carolina, United States of America; 3 Duke Global Health Institute, Duke University, Durham, North Carolina, United States of America; 4 Duke Center for Policy Impact in Global Health, Duke Global Health Institute, Durham, North Carolina, United States of America; 5 Department of Population Health, Duke School of Medicine, Durham, North Carolina, United States of America; 6 Department of Surgery, Duke University School of Medicine, Durham, North Carolina, United States of America; 7 Department of Global Health and Social Medicine, Harvard Medical School, Boston, Massachusetts, United States of America; 8 Mercy Ships, Tyler, Texas, United States of America; McGill University, CANADA

## Abstract

Over 1.7 billion children lack access to surgical care, mostly in low- and middle-income countries (LMICs), with substantial risks of catastrophic health expenditures (CHE) and impoverishment. Increasing interest in reducing out-of-pocket (OOP) expenditures as a tool to reduce the rate of poverty is growing. However, the impact of reducing OOP expenditures on CHE remains poorly understood. The purpose of this study was to estimate the global impact of reducing OOP expenditures for pediatric surgical care on the risk of CHE within and between countries. Our goal was to estimate the impact of reducing OOP expenditures for surgical care in children for 149 countries by modeling the risk of CHE under various scale-up scenarios using publicly available World Bank data. Scenarios included reducing OOP expenditures from baseline levels to paying 70%, 50%, 30%, and 10% of OOP expenditures. We also compared the impact of these reductions across income quintiles (poorest, poor, middle, rich, richest) and differences by country income level (low-income, lower-middle-income, upper-middle-income, and high-income countries).Reducing OOP expenditures benefited people from all countries and income quintiles, although the benefits were not equal. The risk of CHE due to a surgical procedure for children was highest in low-income countries. An unexpected observation was that upper-middle income countries were at higher risk for CHE than LMICs. The most vulnerable regions were Africa and Latin America. Across all countries, the poorest quintile had the greatest risk for CHE. Increasing interest in financial protection programs to reduce OOP expenditures is growing in many areas of global health. Reducing OOP expenditures benefited people from all countries and income quintiles, although the benefits were not equal across countries, wealth groups, or even by wealth groups within countries. Understanding these complexities is critical to develop appropriate policies to minimize the risks of poverty.

## Introduction

Access to quality, timely, and affordable surgical care for children has important economic and health implications on the individual, community, and macroeconomic levels [1–10]. In fact, the “extricable links between health, wealth, and profits” collection of articles at BMJ has highlighted that the greatest gains in economic growth through investment in health is by investing in non-communicable diseases, such as surgical diseases [11]. Despite this, investment in the scale up of children’s surgical services has historically been low in the global health agenda. Over 1.7 billion children do not have access to surgical care, mostly in low- and middle-income countries (LMICs), with substantial risks of catastrophic expenditures and impoverishment [12]. Improving access for these children by reducing financial barriers is needed, with the greatest need among rural communities [13–25].

Equitable access to surgical care for children supports long-term economic development and productivity by preventing premature death and reducing the disabilities that arise from untreated surgical conditions [26]. However, the need for surgical care puts many households in LMICs at risk for impoverishment [27]. To achieve the United Nations (UN) Sustainable Development (SDG) Goal 3, including the provision of universal health coverage (UHC) and financial risk protection, greater attention must be paid to the direct and indirect financial protection mechanisms for surgical care [25, 28]. At the macroeconomic level, surgical care has shown to be one of the most cost-effective interventions in global health, with a 1:10 cost–benefit return on investment ratio [29–32]. At the microeconomic level, investments in surgical care protect families from falling into poverty[19, 30, 33]. Although the macroeconomic and microeconomic benefits of surgical care are well defined, continued reliance on out-of-pocket (OOP) expenditures to support surgical care leads to high rates of catastrophic expenditures and impoverishment [33].

Increasing interest in initiatives to reduce OOP health expenditures as a tool to reduce the rate of poverty is growing in many areas of health care. However, the impact of reducing OOP expenditures on catastrophic health expenditures (CHE) and impoverishment related to surgical care remains poorly understood. Previous studies have shown that understanding the impact of reducing OOP expenditures by socioeconomic status is crucial to design tailored interventions within each country [27, 33–35]. Our previous work described the impact of reducing OOP expenditures for children’s surgical care on the risks of impoverishment across wealth quintiles in Somaliland [27, 33]. Our findings showed that the greatest benefits of financial risk protection were limited to the most wealthy populations and, like previous studies have found, more comprehensive health coverage frameworks, including reduction in direct and non-direct medical expenses, were needed to protect the poorest communities [33–35].

The purpose of this current study was to estimate the impact of reducing OOP expenditures for pediatric surgical care on the global risk of CHE across wealth quintiles and between countries. This data may provide valuable information for the development of financial protection mechanisms within UHC programs to reduce the risks of CHE from health expenditures on a global scale.

## Methods

The goal of this cross-sectional global economic evaluation was to estimate the impact of reducing OOP expenditures for surgical care in children for 149 countries by modeling the risk of CHE under various scale-up scenarios (see **S1 Table**). Scenarios included reducing OOP expenditures from baseline levels to paying 70%, 50%, 30%, and 10% of OOP expenditures. In addition, we compared the impact of these reductions across five income quintiles (poorest, poor, middle, rich, richest) and differences by country income level (low income, low-middle income, upper middle income, and high income). This study follows the Consolidated Health Economic Evaluation Reporting Standards (CHEERS) (see **S1 Checklist**).

### Data input parameters

#### Country-level data

We classified each country according to the World Bank income classification level. Of the 149 total countries, 20 were low-income countries (LICs), 49 were lower-middle income countries (LMICs), 39 were upper-middle countries (UMICs), and 41 were high-income countries (HICs) [36]. According to the World Health Organization (WHO) regions, there were 41 countries from the Africa region (AFR), 22 countries from the Americas (AMR) region, 12 countries from the Eastern Mediterranean (EMR) region, 49 countries from the Europe (EUR) region, 10 countries from the South-East Asia (SEAR) region, and 15 countries from the Western Pacific (WPR) region [37].

Population estimates, Gini indices, gross domestic product (GDP) per capita, household expenditures per capita, and the percent urban population for each country were obtained from the World Bank Data [38, 39]. The rate of poverty at $1.90 per day and at $3.20 per day as a percentage of the population was obtained from the World Bank Data [38]. The poverty gap at $2.15 a day was obtained from the World Bank. Data unless otherwise noted is located in the supplementary material [40]. Data on poverty gaps at $2.15 a day were unavailable for Afghanistan, Antigua and Barbuda, Bahamas, Barbados, and Cambodia, and the national poverty line or $1.90 a day was used instead [41–46]. We obtained all data for the year 2020 (see **S2 Table** for variable definitions). In cases where data on these variables were not available for 2020, we obtained data from the most recent year available.

#### Income quintile estimates

Country-specific GDP per capita and income quintiles were calculated in the following steps. For each income quintile, assuming a gamma distribution, we selected the correct shape for each country’s Gini index [47]. For each candidate GDP per capita, we created 25 income distributions, mean poverty head counts, defined as headcount ratio is the percentage of the population living below the national poverty line(s), and poverty gaps, defined as the ratio by which the mean income of the poor falls below the poverty line. We selected the GDP per capita which approximated the target poverty head count, and compared it to the appropriate gap index for accuracy. For several countries, the estimates were not consistent to the headcount proportions or poverty gaps. We did the same procedure with household expenditure per capita (HEpc) and compared the results to the poverty gap and headcount proportions. In this case, the models were more consistent with the poverty gap and headcount proportions, displaying calibration and accuracy in our estimates. We then found the quintiles for each gamma distribution. We created new gamma distributions, parameterized with the same shape (Gini), with the means pegged to 0.1, 0.3, 0.5, 0.7, and 0.9 times the HEpc from the last step. Lastly, we reparameterized a gamma distribution with the mean income at each value. We then tested how well the model estimates fit the country’s poverty headcount and poverty gap proportions and found the model to be better calibrated. Thus, we used HEpc for our estimate of household income.

The parameterization of the distribution, given any HEpc and Gini index, is given in Eq 1, where *OOP* is the proportion of healthcare expenditure that is paid out-of-pocket, *c* is healthcare expenditure, *t* is the threshold for catastrophic expenditure, *alpha* is the shape parameter, which is proportional to the Gini index, and *beta* is the scale parameter, which is proportional to HEpc [47].


$$E(CE)=\frac{N}{\Gamma (\alpha )\beta^{\alpha }}\int_{0}^{\frac{OOP.c}{t}}y^{\alpha -1}e^{-\frac{y}{\beta }}dy$$ Eq 1

#### Calculation of direct costs

Data on actual costs of individual surgical procedures for children in LMICs are scarce in the literature and vary widely by setting and type of hospital (for-profit, non-profit, government) [48, 49]. In the absence of pediatric-surgery specific cost data, we used proxy measures of costs related to surgical procedures in adults for a range of countries from Gibbons and colleagues [50] as well as data from the Lancet Commission on Global Surgery for a Caesarean section ($179 for low-income countries and $219 for high-income countries) [29, 49]. We used the midpoint unit cost between low-income countries and lower middle-income countries as our baseline estimate of $200 per pediatric surgical procedure [49]. To assess face validity of the $200 unit cost, we compared the estimated costs of other common surgical procedures from a previous systematic review [18, 51]. In the review, the costs ranged from $130-$480 for the most common procedures, excluding the high costs associated with neurological conditions ($608).

### Outcome definitions: Risk of catastrophic expenditures

Our main outcome was CHE due to surgery [47]. CHE was defined as OOP expenditures of greater than 10% of the patient’s estimated income based on World Health Organization (WHO) standards [52, 53]. To calculate CHE, we used the following formula in Eq 2, where *c* represents the OOP expenditures for the procedure, *t* is the chosen threshold level, *y* is the GDP per capita.


c>=ty Eq 2

### Data analysis

We used the catastrophic and impoverishing expenditure tool developed by Shrime *et al*. [47] to analyze the population-level risk of CHE due to direct medical costs by region, rurality, and between income quintiles. We evaluated the impact of reducing OOP expenditures to 70%, 50%, 30%, and 10% by income quintile and region. Descriptive statistics were used to analyze the data collected and compared by income quintile and region. We analyzed the data using SAS Software 9.4 (Cary, NC), R V3.0 (R v3·0 (www.r-project.org), Microsoft Excel 2018 (Redmond, WA).

## Results

**Fig 1:**
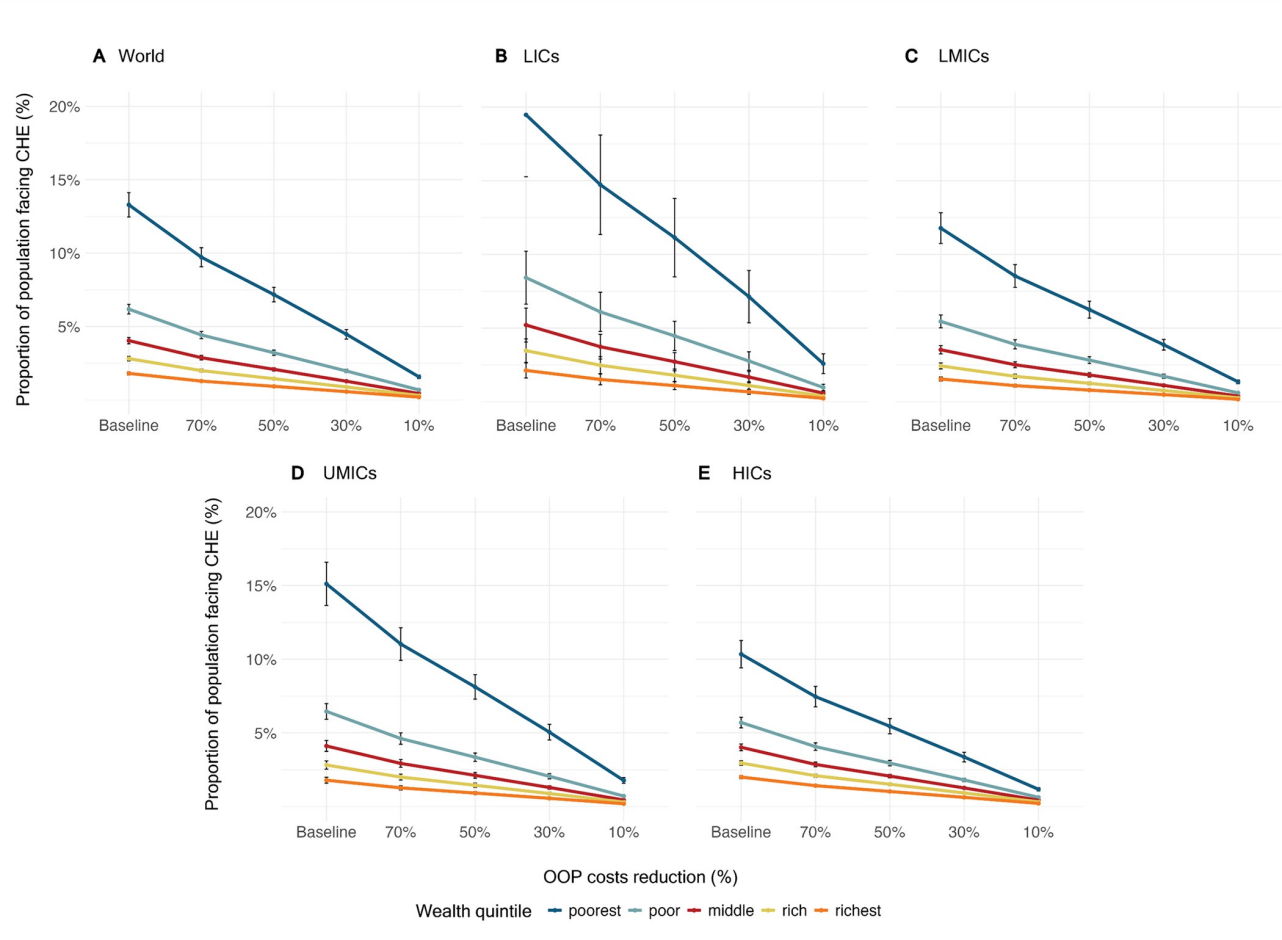
Impact of reducing out-of-pocket costs for pediatric surgery on catastrophic health expenditure by income quintile and World Bank income classification. Error bars represent standard error of the mean.

We found that the risk of CHE due to a surgical procedure for children out of all healthcare costs was highest in LICs or among the poorest income quintile within countries across all income levels at baseline (**Fig 1**). Reducing OOP expenditures benefited all countries and income quintiles, although the benefits were not equal. The risk in low-income countries (LICs) was 7.7% at baseline, compared to 4.9%, 6.1%, and 5.0% in LMICs, UMICs, and HICs respectively (**S3 Table**). Comparing by income quintiles, the risk was the highest for the poorest quintiles in each country income-level group, with the highest overall for LICs (19.5% in LICs vs 11.8% in LMICs, 15.1% in UMICs, and 10.3% in HICs). The risk for the richest quintile in each country group was 2.0% overall (2.1% in LICs, 1.5% in LMICs, 1.8% in UMICs, and 2.0% in HICs). Although the risk was similar in the richest quintile in each country income-level group, the greatest difference at baseline between the richest and poorest quintiles was in LICs, with a difference of 17.4 percentage points (19.5% risk in poorest quintile—2.1% risk in richest quintile), compared to a difference of 10.5 percentage points in LMICs, 13.5 percentage points in UMICs, and 8.3 percentage points in HICs. The risk of CHE was reduced for all quintile levels and country income-level groups with the greatest reductions seen at the 10% OOP level.

**Fig 2:**
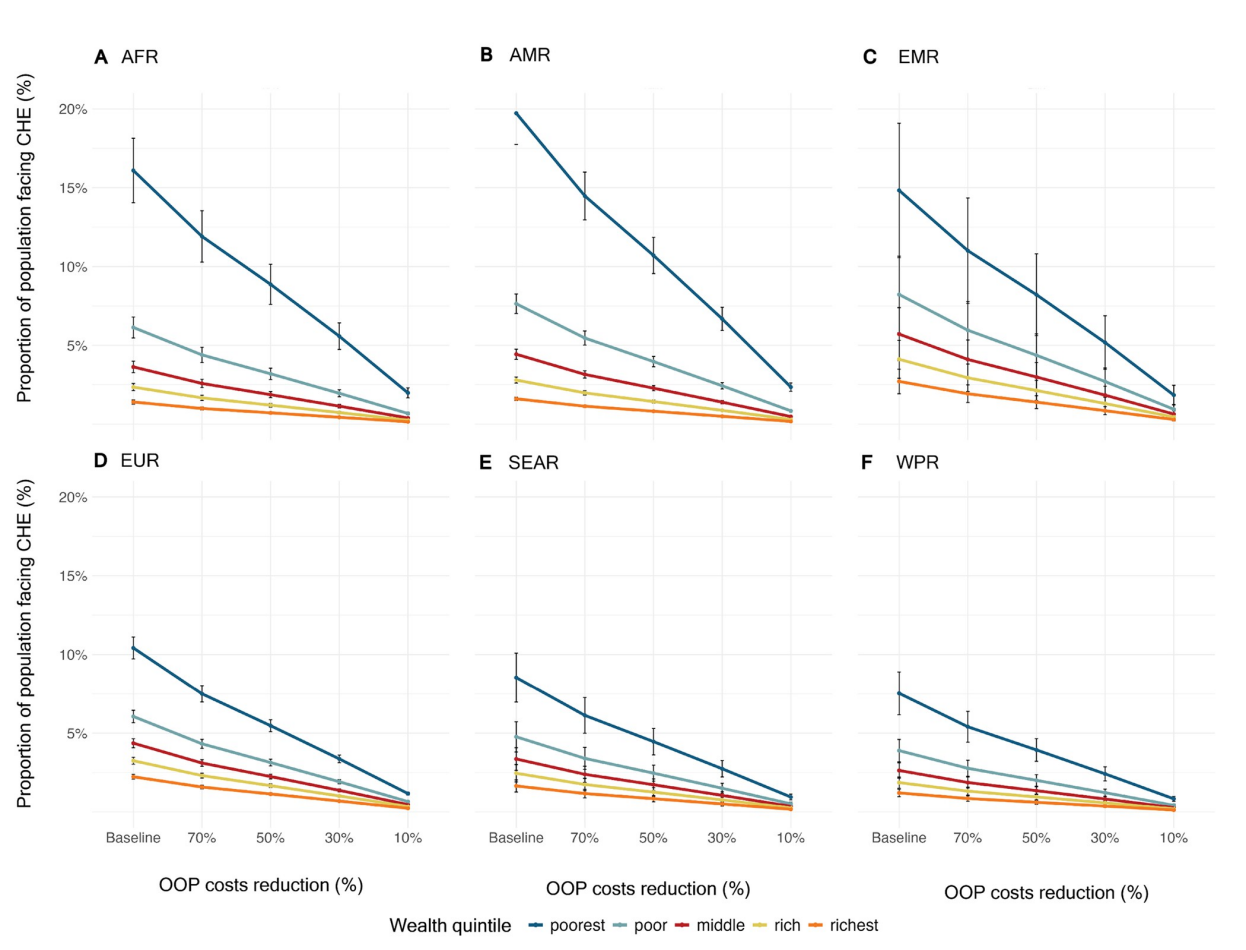
Impact of reducing out-of-pocket costs for pediatric surgery on catastrophic health expenditure by income quintile and World Health Organization regions. Error bars represent standard error of the mean.

At baseline, the AMR and EMR regions had the highest risk of catastrophic health expenditures (7.2% and 7.1%, respectively), followed by the AFR regions (5.9%) (**Table 1**). When stratified by income quintile, the risks for the poorest quintile nearly tripled for the AFR, AMR, and EMR regions and doubled for the EUR, SEAR, and WPR regions, compared with their baseline risks (**Fig 2**). The risk for each region was concentrated within the poorest quintile, with nearly triple the risks compared to the next income quintile, the poor quintile. For example, the risks for the AFR region were 6.1% in the poor quintile, but 16.1% in the poorest quintile. Notably, the risks for the poor quintile mirrored the overall risk for each region, showing that the risk was largely concentrated in the poorest quintile. The risk was reduced in each OOP scenario, with the most notable reduction occurring in the poorest quintile.

**Table 1:** Proportion of population at risk of catastrophic health expenditure upon out-of-pocket costs reduction for pediatric surgery by income quintile and WHO region.

WHO Region	Wealth Quintile	Baseline	70%	50%	30%	10%
AFR	**Overall**	**0.059**	**0.043**	**0.032**	**0.020**	**0.007**
	poorest	0.161	0.119	0.089	0.056	0.020
	poor	0.061	0.044	0.032	0.020	0.007
	middle	0.036	0.026	0.019	0.011	0.004
	rich	0.024	0.017	0.012	0.007	0.003
	richest	0.014	0.010	0.007	0.004	0.002
AMR	**Overall**	**0.072**	**0.052**	**0.038**	**0.024**	**0.008**
	poorest	0.197	0.145	0.107	0.067	0.024
	poor	0.076	0.055	0.040	0.024	0.008
	middle	0.044	0.032	0.023	0.014	0.005
	rich	0.028	0.020	0.014	0.009	0.003
	richest	0.016	0.011	0.008	0.005	0.002
EMR	**Overall**	**0.071**	**0.052**	**0.038**	**0.024**	**0.008**
	poorest	0.148	0.110	0.082	0.052	0.018
	poor	0.082	0.060	0.044	0.027	0.009
	middle	0.057	0.041	0.030	0.018	0.006
	rich	0.041	0.029	0.021	0.013	0.005
	richest	0.027	0.019	0.014	0.009	0.003
EUR	**Overall**	**0.053**	**0.038**	**0.027**	**0.017**	**0.006**
	poorest	0.104	0.075	0.055	0.034	0.012
	poor	0.061	0.043	0.031	0.019	0.007
	middle	0.044	0.031	0.022	0.014	0.005
	rich	0.033	0.023	0.017	0.010	0.004
	richest	0.022	0.016	0.011	0.007	0.002
SEAR	**Overall**	**0.042**	**0.030**	**0.022**	**0.013**	**0.005**
	poorest	0.085	0.061	0.045	0.027	0.010
	poor	0.048	0.034	0.025	0.015	0.005
	middle	0.034	0.024	0.017	0.011	0.004
	rich	0.025	0.017	0.013	0.008	0.003
	richest	0.017	0.012	0.008	0.005	0.002
WPR	**Overall**	**0.034**	**0.024**	**0.018**	**0.011**	**0.004**
	poorest	0.075	0.054	0.039	0.024	0.008
	poor	0.039	0.028	0.020	0.012	0.004
	middle	0.026	0.019	0.014	0.008	0.003
	rich	0.019	0.013	0.010	0.006	0.002
	richest	0.012	0.009	0.006	0.004	0.001

AFR = Africa region, AMR = Americas region, EMR = Eastern Mediterranean region, EUR = Europe region, SEAR = South-East Asia region, WPR = Western Pacific region.

**Fig 3:**
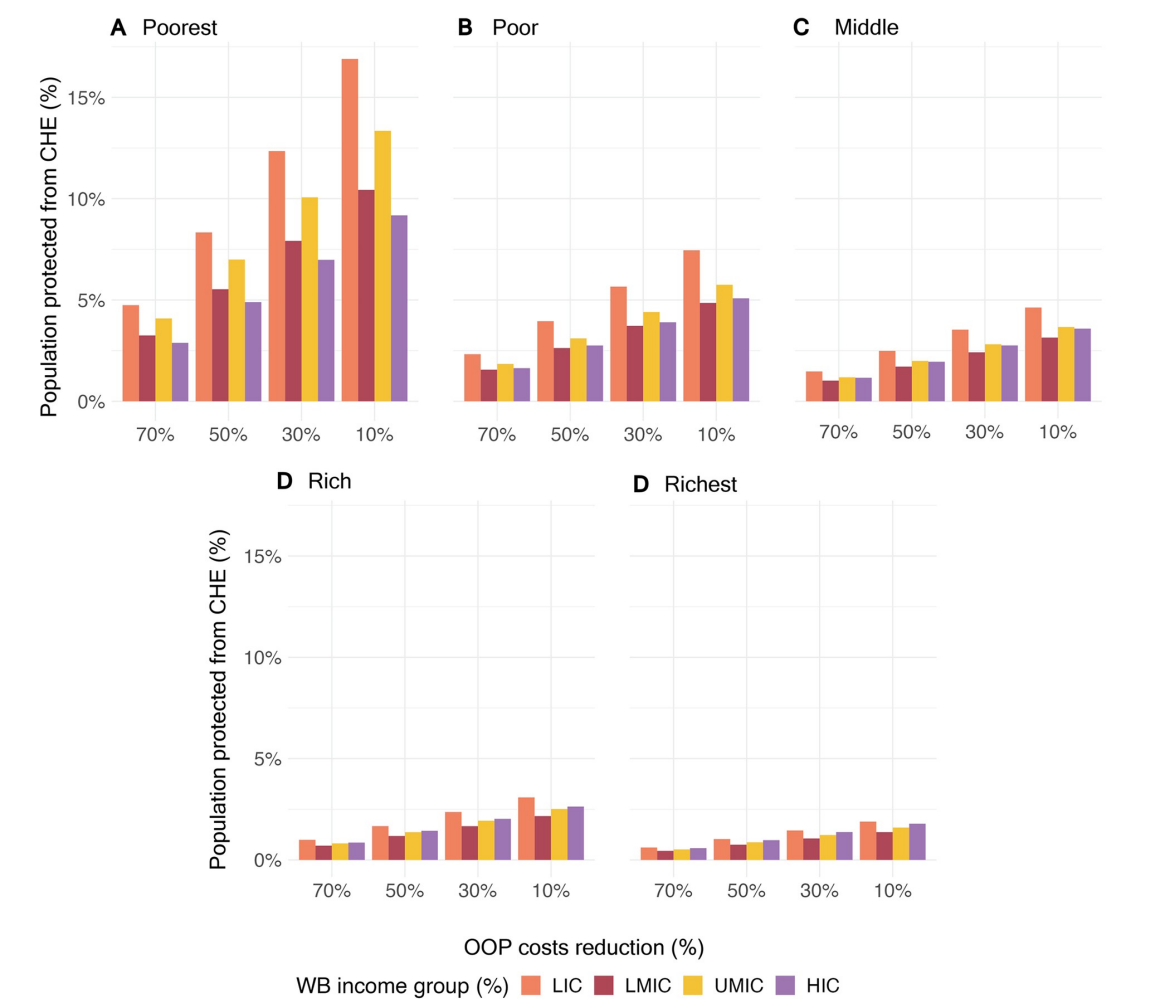
Proportion of population protected from catastrophic health expenditure upon out-of-pocket costs reduction for pediatric surgery. Error bars represent standard error of the mean.

The proportions of the population protected from CHE if the OOP expenditures were the highest for LICs at all reduction levels (**Fig 3, see S4 Table** for details). LICs saw the greatest overall benefits, ranging from 2.0% if the OOP expenditures were reduced to 70% to 6.8% if the OOP expenditures were reduced to 10%. UMICs saw the next greatest overall benefits, ranging from 1.7% if the OOP expenditures were reduced to 70%, and 5.4% if the OOP expenditures were reduced to 10%. When stratified by income quintile, the greatest benefit was in the poorest quintile and when OOP expenditures were reduced to 10%. For example, 16.9% of the population living in the poorest quintile in LICs would be protected from catastrophic health expenditure if the OOP expenditures were reduced to 10%, compared to only protecting 4.7% if the costs were reduced to 70% or 8.3% if the costs were reduced to 50%.

The proportion of the population protected from risk of CHE was highest in the AMR, EMR, and AFR regions across all income quintiles and reduction scenarios (**Table 2**). Notably, the reduction was concentrated in the poorest quintiles for all regions. For example, reducing the OOP expenditures to 50% in the AFR region protected 1.8% and 2.9% of the population in the middle or poor quintiles, respectively, but 7.2% in the poorest quintile. Similar trends are seen in the other regions. The greatest proportion protected from CHE occurred when OOP expenditures were reduced to 10% for the poorest quintile in the AMR region (17.4%), followed by the same quintile and scenario in the AFR region (14.1%), and in the EMR region (13.0%).

**Table 2:** Proportion of population protected from catastrophic health expenditure upon out-of-pocket costs reduction for pediatric surgery by income quintile and WHO region.

WHO Region	Income quintile	70%	50%	30%	10%
AFR	**Overall**	**0.016**	**0.028**	**0.040**	**0.052**
	poorest	0.042	0.072	0.105	0.141
	poor	0.017	0.029	0.042	0.055
	middle	0.010	0.018	0.025	0.032
	rich	0.007	0.011	0.016	0.021
	richest	0.004	0.007	0.010	0.013
AMR	**Overall**	**0.020**	**0.034**	**0.049**	**0.064**
	poorest	0.053	0.090	0.130	0.174
	poor	0.022	0.037	0.052	0.068
	middle	0.013	0.022	0.030	0.040
	rich	0.008	0.014	0.019	0.025
	richest	0.005	0.008	0.011	0.014
EMR	**Overall**	**0.019**	**0.033**	**0.047**	**0.063**
	poorest	0.038	0.066	0.097	0.130
	poor	0.023	0.039	0.055	0.073
	middle	0.016	0.027	0.039	0.051
	rich	0.012	0.020	0.028	0.037
	richest	0.008	0.013	0.019	0.024
EUR	**Overall**	**0.015**	**0.025**	**0.036**	**0.047**
	poorest	0.029	0.049	0.070	0.092
	poor	0.017	0.029	0.041	0.054
	middle	0.013	0.021	0.030	0.039
	rich	0.009	0.016	0.022	0.029
	richest	0.007	0.011	0.015	0.020
SEAR	**Overall**	**0.012**	**0.020**	**0.028**	**0.037**
	poorest	0.024	0.041	0.058	0.076
	poor	0.014	0.023	0.033	0.042
	middle	0.010	0.016	0.023	0.030
	rich	0.007	0.012	0.017	0.022
	richest	0.005	0.008	0.011	0.015
WPR	**Overall**	**0.010**	**0.017**	**0.023**	**0.031**
	poorest	0.021	0.036	0.051	0.067
	poor	0.011	0.019	0.027	0.035
	middle	0.008	0.013	0.018	0.024
	rich	0.005	0.009	0.013	0.017
	richest	0.004	0.006	0.008	0.011

AFR = Africa region, AMR = Americas region, EMR = Eastern Mediterranean region, EUR = Europe region, SEAR = South-East Asia region, WPR = Western Pacific region.

## Discussion

Our paper aligns strongly with the landmark collection of papers at BMJ on the links between health, wealth, and profits. Children’s surgical diseases make up a large part of the growing non-communicable diseases in the world, particularly in low-income countries where investment in health and economic development are needed. Increasing interest in financial protection programs to reduce OOP expenditures is growing in many areas of global health. However, these programs are difficult to implement and have variable impact on clinical and financial outcomes. In particular, the impact of OOP expenditures on CHE related to surgical care remains poorly understood. The purpose of this current study was to estimate the impact of reducing OOP expenditures for pediatric surgical care globally. We found that reducing OOP expenditures benefited all countries and income quintiles, although the benefits were not equal across countries, wealth groups, or even by wealth groups within countries. Understanding these complexities is critical to develop appropriate policies to minimize the risks of poverty related to surgical care.

Overall, we found that LICs and the poorest quintiles among all countries are at the highest risk of CHE due to OOP expenditures for pediatric surgical care. A previous study by Shrime et al. demonstrated similar findings, with individuals within the poorest income quintiles at the highest risk of CHE for surgical care [27]. Interestingly, we found that UMICs are at higher risk for CHE at baseline and benefited greater from OOP expenditures reductions compared to LMICs. Existing literature examining financial risk protection for both adult and pediatric surgical care suggests that as a country’s economic status or GDP increases, the cost of care may also increase, potentially leaving a higher number of people without sufficient financial protection, despite having greater access to insurance [27]. This may be due to existing income inequality as well as unequal increases in household income, unaffordability of insurance programs, and lack of proper coverage within insurance schemes, compared to the rate of economic development within the country. Lastly, we found that while HICs also benefited from reductions in OOP expenditures for pediatric surgery, the benefit for these countries was not as significant compared to LICs, LMICs, and UMICs.

Although all WHO regions showed increased protection from decreased OOP expenditures for pediatric surgical care, AMR, EMR, and AFR regions have the highest risk for CHE at baseline and thus experience the greatest protection from CHE overall. We suspect that the AMR region may experience large variability in access to care as well as financial risk protection for surgery at the country level [47, 54]. This variation in supply of appropriate surgical services may impact costs at the household level. For example, areas with limited resources necessary to provide surgical care may experience a particularly high cost of surgery compared to other countries in the same region.

Importantly, we found that lowering OOP expenditures benefits people in poorest quintiles around the world, regardless of the country’s overall income level. The poorest quintiles in the poorest countries suffer a double burden of poverty at the individual and country-level, and providing financial risk protection directly addresses this challenge. Financial risk protection schemes should be directed at all poor quintiles, even in high-income countries. An analysis of 24 European countries found that catastrophic health spending is mainly concentrated in the poorest households, even though the OOP expenditures are higher in the richer quintiles meaning the poorer households are at greater risk despite lower OOP expenditures [55]. Similar results are found in the United States, with the risk of CHE higher in low-income communities [56]. The within-country variability of the risk of CHE is a strong motivator for researchers to not only compare country income levels, but also income quintiles within the country.

From a policy perspective, these findings suggest that financial protection programs are needed for poorer communities regardless of the country they live in, particularly for households in the poorest quintiles. Investing in health among poor families not only benefits individuals, but the societies they live in. Reducing the risk of CHE encourages health seeking behaviors improving health outcomes, and subsequently improves economic outcomes [57, 58]. The link between poverty reduction, including reducing health-related OOP expenditures should be highlighted to policy makers working towards reducing income inequality in their country. Our study found that reducing OOP expenditures related to pediatric surgical care is a strategic opportunity towards that goal. Our model suggests that the risk of CHE is higher for LICs and LMICs compared to UMICs and HICs, and among the AMR, EMR, and AFR regions. With the emphasis in the United Nations SDG 1 of ending poverty in all its forms everywhere, SDG 3 of ensuring healthy lives and promoting well-being for all, and the WHO goals of achieving 80% essential healthcare service coverage and 100% protection from catastrophic and impoverishing expenditures within UHC schemes by 2030 [59–61], our data suggests that surgery should be included to achieve those goals. As current financial schemes do not match the need for financial risk protection for surgical care, there is a need for scaling up of strategic financial protection, especially in LICs, LMICs and AFR [25]. Our previous research found that LICs and LMICs are more likely to include pediatric surgery in their national health policies, strategies, and plans (NHPSPs) compared to UMICs and HICs [25]. Globally, less than 18% of countries across incomes and regions include pediatric surgical coverage in NHPSPs, and surgical care remains excluded from most global funding priorities. For instance, in 2018 most of the global health funding of USD 38.9 billion was allocated to infectious diseases (39%), child health and maternal health (32%), and health system strengthening (14%), with only 2% being allocated to non-communicable diseases, including surgical care [62].

While the need to reduce OOP expenditures related to surgical care is evident, the outstanding question relates to how countries can achieve this goal. The policy path to incorporate surgical care into UHC programs can be explored using the three dimensions of the WHO UHC cube [33, 63]. The *depth* dimension denotes the range of surgical services covered, the *breath* dimension represents who is covered, and the *height* dimension denotes the proportion of surgical costs covered. While each country might be at a different stage on this path, these dimensions provide a framework to guide policy expansion in each country.

As a first step in the *depth* of surgical coverage, focus should be put on defining an essential and basic emergency surgical package [64]. Incorporating this essential surgical package into existing healthcare packages, such as maternal and child health plans or HIV test-and-treat programs, can build upon the progress already made in these sectors in LMICs [65–67]. Particular attention must be put on surgical procedures that avert a significant portion of the health burden in the pediatric population. Inguinal hernia repair, congenital heart surgery and cleft lip/palate repair are examples of known “essential pediatric surgical procedures”, given the economic value they offer in preventing health burden [18]. Second, to make progress in the *breath* dimension, governments usually start by providing coverage to certain groups such as formal sector workers [68]. However, this often delays the provision of financial protection to the poorest, most vulnerable groups. Prioritizing equity in health financing reforms such as pooling mechanisms ensures protection across all income levels [69]. Third, the *height* dimension, the proportion of OOP costs for surgical services, is one of the main predictors to CHE for families in LMICs [33]. How to reduce these costs relates to the overarching question of how to finance UHC overall.

The UHC action agenda presented in the 2023 UN High Level Meeting, advocates for increased public spending on health and the use of pooled funds to strengthen financial protection, particularly for the most vulnerable populations [70]. A specific focus is given to increased domestic resource mobilization where UHC can be financed through various mechanisms. Tax-financed systems have a significant revenue-raising and pooling potential due to their large tax base [70]. A health care model that relies on taxation is the Beveridge model implemented in HICs like the United Kingdom and Norway [71, 72]. Next, social and national health insurance schemes are financed through contributions to social insurance funds and are often complemented by public subsidies [68, 73]. For example, the Bismarck model in Germany and Japan functions through social insurance funds and Taiwan and South Korea use government-run insurance programs [72, 73]. Because these mechanisms offer some level of pooling and cross subsidization, whether used independently or combined, they have been recognized as appropriate mechanisms to achieve UHC [70]. Ultimately, the goal is to move away from OOP models and small, fragmented health financing pools [74–76]. The WHO advises countries to move towards larger pools where the financial risk can be spread across the population [74]. This way, essential health services, including surgical care, can be distributed across the entire nation and equitable access to these services is possible.

Regarding surgical care specifically, a National Surgical, Obstetric and Anesthesia Plan (NSOAP) is a valuable tool to evaluate the technical, political, and financial feasibility of surgical expansion at the country level and many LMICs such as Tanzania, Ethiopia, and Rwanda already developed their own [29, 77]. As a result, each country can create its own strategic roadmap to leverage existing healthcare packages or financial protection mechanisms, with the aim of reducing high OOP expenditures for families seeking surgical care.

We chose one price point per surgical procedure for all countries rather than separate price points per income level of the country. The $200 includes direct medical costs for preoperative evaluation, hospital care, and routine post-operative care as well as the recurrent costs of running a surgical service such as salaries, utilities, equipment, medical supplies and medicines. Although the approximation of $200 could differ by income level of the country, we do not expect it to affect the results in substantial ways given the $200 is a conservative proxy estimate. In fact, our results might be attenuated towards the null given the conservative estimate we used. In addition, the $200 estimate is based on the Lancet Commission on Global Surgery’s estimate of average surgical procedure for LICs and LMICs and was similar to most other common pediatric surgical procedures in our systematic review [18]. Thus, if the cost estimate is inaccurate for a certain income level, it is likely accurate for LICs and LMICs and underestimated for HICs. Finally, our model is agnostic about the source of funds for OOP reduction. Our study has several limitations common to economic modeling studies. First, our modeling was based on available public data, and thus was limited to countries with the data available through the World Bank Repository for our primary input parameter of HEpc. Of the 189 World Bank countries, 40 were excluded due to unavailable data limiting our analyses to 149 countries. Of the 40 excluded, 80% were from UMICs or HICs, improving the model’s interpretability to the fuller datasets from LICs and LMICs. Moreover, the included countries account for about 95% of the world’s population, capturing a substantial majority of the global population [78]. Second, for some countries, data was only available for older years which means that for these cases, the model may not capture the country’s complete economic development. Although this may not depict the country’s current economic status, we do not expect major economic changes to occur within a few years. Third, our analyses did not address health impacts, so results must be interpreted in that light. Depending on the country, resource allocation and prioritization of resources may be driven by health outcomes, financial-risk protection outcomes, other factors, or a combination of several factors. Third, we did not account for disease burden, health seeking behaviors, and insurance coverage. While this does not influence our estimates, it will be important for policy makers that need actual estimates of the cost (funds needed) to reduce OOP by the desired percentage. Policy implications will be different if countries choose to achieve OOP reduction through public financing versus social health insurance, or development assistance for health.

Moving forward, the inclusion of financial risk protection for surgical care for children under a broad umbrella of improved financing for child health systems will leverage funding allocated to this population and improve children’s health globally [30]. As well, consideration of innovative funding programs, where monetary commitments are typically linked to health system inputs or outputs, may offer novel alternatives to traditional funding mechanisms for surgical care [62].

## Supporting information

S1 Checklist Research checklist: Consolidated Health Economic Evaluation Reporting Standards (CHEERS) 2022 check list. (DOCX)

S1 Table List of countries. (DOCX)

S2 Table Variable definitions. (DOCX)

S3 Table Proportion of population at risk of catastrophic health expenditure upon out-of-pocket costs reduction for pediatric surgery by income quintile and World Bank income. (DOCX)

S4 Table Proportion of population protected from risk of catastrophic health expenditure upon out-of-pocket costs reduction for pediatric surgery by income quintile and World Bank income. (DOCX)
