# Supplementary material for: Modeling the global impact of reducing out-of-pocket costs for children’s surgical care
Source: PLOS Glob Public Health. 2024 Jan 26;4(1):e0002872. doi: 10.1371/journal.pgph.0002872 (PMC10817198; doi:10.1371/journal.pgph.0002872)
Supplement: S1 Table — (DOCX) [file pgph.0002872.s002.docx]

**S1 Table.** List of countries.

| Albania |
| --- |
| Algeria |
| Angola |
| Argentina |
| Armenia |
| Australia |
| Austria |
| Azerbaijan |
| Bangladesh |
| Belarus |
| Belgium |
| Belize |
| Benin |
| Bhutan |
| Bolivia |
| Bosnia and Herzegovina |
| Botswana |
| Brazil |
| Bulgaria |
| Burkina Faso |
| Burundi |
| Cabo Verde |
| Cameroon |
| Canada |
| Central African Republic |
| Chad |
| Chile |
| China |
| Colombia |
| Comoros |
| Congo, Dem. Rep. |
| Congo, Rep. |
| Costa Rica |
| Cote d'Ivoire |
| Croatia |
| Cyprus |
| Czech Republic |
| Denmark |
| Djibouti |
| Dominican Republic |
| Ecuador |
| Egypt, Arab Rep. |
| El Salvador |
| Estonia |
| Eswatini |
| Ethiopia |
| Fiji |
| Finland |
| France |
| Gabon |
| Gambia, The |
| Georgia |
| Germany |
| Ghana |
| Greece |
| Guatemala |
| Guinea |
| Guinea-Bissau |
| Haiti |
| Honduras |
| Hungary |
| Iceland |
| India |
| Indonesia |
| Iran, Islamic Rep. |
| Iraq |
| Ireland |
| Israel |
| Italy |
| Jamaica |
| Japan |
| Jordan |
| Kazakhstan |
| Kenya |
| Kiribati |
| Korea, Rep. |
| Kyrgyz Republic |
| Lao PDR |
| Latvia |
| Lebanon |
| Lesotho |
| Lithuania |
| Luxembourg |
| Madagascar |
| Malaysia |
| Maldives |
| Mali |
| Malta |
| Mauritania |
| Mauritius |
| Mexico |
| Moldova |
| Mongolia |
| Montenegro |
| Morocco |
| Mozambique |
| Myanmar |
| Namibia |
| Nepal |
| Netherlands |
| Nicaragua |
| Niger |
| Nigeria |
| North Macedonia |
| Norway |
| Pakistan |
| Panama |
| Paraguay |
| Peru |
| Philippines |
| Poland |
| Portugal |
| Romania |
| Russian Federation |
| Rwanda |
| Samoa |
| Senegal |
| Serbia |
| Seychelles |
| Sierra Leone |
| Slovak Republic |
| Slovenia |
| Solomon Islands |
| South Africa |
| Spain |
| Sri Lanka |
| Sudan |
| Sweden |
| Switzerland |
| Syrian Arab Republic |
| Tajikistan |
| Tanzania |
| Thailand |
| Timor-Leste |
| Togo |
| Tonga |
| Tunisia |
| Turkey |
| Uganda |
| Ukraine |
| United Arab Emirates |
| United Kingdom |
| United States |
| Uruguay |
| Uzbekistan |
| Vanuatu |
| Vietnam |
| Zambia |
| Zimbabwe |
