# Supplementary material for: Modeling the global impact of reducing out-of-pocket costs for children’s surgical care
Source: PLOS Glob Public Health. 2024 Jan 26;4(1):e0002872. doi: 10.1371/journal.pgph.0002872 (PMC10817198; doi:10.1371/journal.pgph.0002872)
Supplement: S2 Table — (DOCX) [file pgph.0002872.s003.docx]

**S2 Table.** Variable definitions

| Variable | Source | Definition |
| --- | --- | --- |
| Population | World Bank Open Data | "Total population is based on the de facto definition of population, which counts all residents regardless of legal status or citizenship. The values shown are midyear estimates." |
| Gini index | World Bank Open Data | "Extent to which the distribution of income (or, in some cases, consumption expenditure) among individuals or households within an economy deviates from a perfectly equal distribution. A Lorenz curve plots the cumulative percentages of total income received against the cumulative number of recipients, starting with the poorest individual or household. The Gini index measures the area between the Lorenz curve and a hypothetical line of absolute equality, expressed as a percentage of the maximum area under the line. Thus a Gini index of 0 represents perfect equality, while an index of 100 implies perfect inequality." |
| Household expenditure per capita | World Bank Open Data | "Current expenditures on health per capita in current US dollars. Estimates of current health expenditures include healthcare goods and services consumed during each year." |
| Out-of-pocket expenditure (% of current health expenditure) | World Bank Open Data | "Share of out-of-pocket payments of total current health expenditures. Out-of-pocket payments are spending on health directly out-of-pocket by households." |
| Poverty headcount ratio at $1.90 a day (2011 PPP) (% of population) | World Bank Open Data | "Poverty headcount ratio at $1.90 a day is the percentage of the population living on less than $1.90 a day at 2011 international prices. As a result of revisions in PPP exchange rates, poverty rates for individual countries cannot be compared with poverty rates reported in earlier editions." |
| Poverty headcount ratio at $3.20 a day (2011 PPP) (% of population) | World Bank Open Data | "Poverty headcount ratio at $2.15 a day is the percentage of the population living on less than $2.15 a day at 2017 purchasing power adjusted prices. As a result of revisions in PPP exchange rates, poverty rates for individual countries cannot be compared with poverty rates reported in earlier editions." |
| Poverty gap at $2.15 a day (2017 PPP) (%) | World Bank Open Data | "Poverty gap at $2.15 a day (2017 PPP) is the mean shortfall in income or consumption from the poverty line $2.15 a day (counting the nonpoor as having zero shortfall), expressed as a percentage of the poverty line. This measure reflects the depth of poverty as well as its incidence." |
| Urban population (%) | World Bank Open Data | "Urban population refers to people living in urban areas as defined by national statistical offices. The data are collected and smoothed by United Nations Population Division." |
| GDP per capita | Calculations provided in main manuscript | Steps for calculations provided in methods section. |
| Household expenditure per capita | Calculations provided in main manuscript | Steps for calculations provided in methods section. |
