# Supplementary material for: Modeling the global impact of reducing out-of-pocket costs for children’s surgical care
Source: PLOS Glob Public Health. 2024 Jan 26;4(1):e0002872. doi: 10.1371/journal.pgph.0002872 (PMC10817198; doi:10.1371/journal.pgph.0002872)
Supplement: S3 Table — (DOCX) [file pgph.0002872.s004.docx]

**S3 Table**. Proportion of population at risk of catastrophic health expenditure upon out-of-pocket costs reduction for pediatric surgery by income quintile and World Bank income classification

| **WB Income Group** | **Income quintile** | **Baseline** | **70% OOP reduction** | **50% OOP reduction** | **30% OOP reduction** | **10% OOP reduction** |
| --- | --- | --- | --- | --- | --- | --- |
| **LICs** |  | **0.077** | **0.057** | **0.042** | **0.027** | **0.009** |
|  | poorest | 0.195 | 0.147 | 0.111 | 0.071 | 0.026 |
|  | poor | 0.084 | 0.061 | 0.045 | 0.028 | 0.010 |
|  | middle | 0.052 | 0.037 | 0.027 | 0.017 | 0.006 |
|  | rich | 0.035 | 0.025 | 0.018 | 0.011 | 0.004 |
|  | richest | 0.021 | 0.015 | 0.011 | 0.007 | 0.002 |
| **LMICs** |  | **0.049** | **0.035** | **0.026** | **0.016** | **0.006** |
|  | poorest | 0.118 | 0.085 | 0.063 | 0.039 | 0.013 |
|  | poor | 0.054 | 0.039 | 0.028 | 0.017 | 0.006 |
|  | middle | 0.035 | 0.025 | 0.018 | 0.011 | 0.004 |
|  | rich | 0.024 | 0.017 | 0.012 | 0.008 | 0.003 |
|  | richest | 0.015 | 0.011 | 0.008 | 0.005 | 0.002 |
| **UMICs** |  | **0.061** | **0.044** | **0.032** | **0.020** | **0.007** |
|  | poorest | 0.151 | 0.110 | 0.081 | 0.051 | 0.018 |
|  | poor | 0.065 | 0.046 | 0.034 | 0.021 | 0.007 |
|  | middle | 0.041 | 0.029 | 0.021 | 0.013 | 0.004 |
|  | rich | 0.028 | 0.020 | 0.014 | 0.009 | 0.003 |
|  | richest | 0.018 | 0.013 | 0.009 | 0.006 | 0.002 |
| **HICs** |  | **0.050** | **0.036** | **0.026** | **0.016** | **0.006** |
|  | poorest | 0.103 | 0.075 | 0.055 | 0.034 | 0.012 |
|  | poor | 0.057 | 0.041 | 0.029 | 0.018 | 0.006 |
|  | middle | 0.040 | 0.029 | 0.021 | 0.013 | 0.004 |
|  | rich | 0.030 | 0.021 | 0.015 | 0.009 | 0.003 |
|  | richest | 0.020 | 0.014 | 0.010 | 0.006 | 0.002 |
