# Supplementary material for: Modeling the global impact of reducing out-of-pocket costs for children’s surgical care
Source: PLOS Glob Public Health. 2024 Jan 26;4(1):e0002872. doi: 10.1371/journal.pgph.0002872 (PMC10817198; doi:10.1371/journal.pgph.0002872)
Supplement: S4 Table — (DOCX) [file pgph.0002872.s005.docx]

**S4 Table.** Proportion of population protected from risk of catastrophic health expenditure upon out-of-pocket costs reduction for pediatric surgery by income quintile and World Bank income.

| **WB Income Group** | **Wealth**  **Quintile** | **70% OOP reduction** | **50% OOP reduction** | **30% OOP reduction** | **10% OOP reduction** |
| --- | --- | --- | --- | --- | --- |
| **LICs** |  | **0.020** | **0.035** | **0.051** | **0.068** |
|  | poorest | 0.047 | 0.083 | 0.123 | 0.169 |
|  | poor | 0.023 | 0.040 | 0.057 | 0.075 |
|  | middle | 0.015 | 0.025 | 0.035 | 0.046 |
|  | rich | 0.010 | 0.017 | 0.024 | 0.031 |
|  | richest | 0.006 | 0.010 | 0.015 | 0.019 |
| **LMICs** |  | **0.014** | **0.024** | **0.034** | **0.044** |
|  | poorest | 0.033 | 0.055 | 0.079 | 0.104 |
|  | poor | 0.016 | 0.026 | 0.037 | 0.049 |
|  | middle | 0.010 | 0.017 | 0.024 | 0.031 |
|  | rich | 0.007 | 0.012 | 0.017 | 0.022 |
|  | richest | 0.004 | 0.008 | 0.011 | 0.014 |
| **UMICs** |  | **0.017** | **0.029** | **0.041** | **0.054** |
|  | poorest | 0.041 | 0.070 | 0.101 | 0.133 |
|  | poor | 0.018 | 0.031 | 0.044 | 0.057 |
|  | middle | 0.012 | 0.020 | 0.028 | 0.037 |
|  | rich | 0.008 | 0.014 | 0.019 | 0.025 |
|  | richest | 0.005 | 0.009 | 0.012 | 0.016 |
| **HICs** |  | **0.014** | **0.024** | **0.034** | **0.045** |
|  | poorest | 0.029 | 0.049 | 0.070 | 0.092 |
|  | poor | 0.016 | 0.028 | 0.039 | 0.051 |
|  | middle | 0.012 | 0.020 | 0.028 | 0.036 |
|  | rich | 0.009 | 0.014 | 0.020 | 0.026 |
|  | richest | 0.006 | 0.010 | 0.014 | 0.018 |
